# Rural Food and Physical Activity Assessment Using an Electronic Tablet-Based Application, New York, 2013–2014

**DOI:** 10.5888/pcd12.150147

**Published:** 2015-07-02

**Authors:** Rebecca A. Seguin, Emily H. Morgan, Leah M. Connor, Jennifer A. Garner, Abby C. King, Jylana L. Sheats, Sandra J. Winter, Matthew P. Buman

**Affiliations:** Author Affiliations: Emily H. Morgan, Leah M. Connor, Jennifer A. Garner, Division of Nutritional Sciences, Cornell University, Ithaca, New York; Abby C. King, Department of Health Research and Policy and Stanford Prevention Research Center, Stanford University School of Medicine, Stanford, California; Jylana L. Sheats, Sandra J. Winter, Stanford Prevention Research Center, Stanford University School of Medicine, Palo Alto, California; Matthew P. Buman, School of Nutrition and Health Promotion, Arizona State University, Phoenix, Arizona.

## Abstract

**Introduction:**

A community’s built environment can influence health behaviors. Rural populations experience significant health disparities, yet built environment studies in these settings are limited. We used an electronic tablet-based community assessment tool to conduct built environment audits in rural settings. The primary objective of this qualitative study was to evaluate the usefulness of the tool in identifying barriers and facilitators to healthy eating and active living. The second objective was to understand resident perspectives on community features and opportunities for improvement.

**Methods:**

Participants were recruited from 4 rural communities in New York State. Using the tool, participants completed 2 audits, which consisted of taking pictures and recording audio narratives about community features perceived as assets or barriers to healthy eating and active living. Follow-up focus groups explored the audit experience, data captured, and opportunities for change.

**Results:**

Twenty-four adults (mean age, 69.4 y [standard deviation, 13.2 y]), 6 per community, participated in the study. The most frequently captured features related to active living were related to roads, sidewalks, and walkable destinations. Restaurants, nontraditional food stores, and supermarkets were identified in the food environment in relation to the cost, quality, and selection of healthy foods available. In general, participants found the assessment tool to be simple and enjoyable to use.

**Conclusion:**

An electronic tablet–based tool can be used to assess rural food and physical activity environments and may be useful in identifying and prioritizing resident-led change initiatives. This resident-led assessment approach may also be helpful for informing and evaluating rural community-based interventions.

## Introduction

Physical inactivity and poor diet are 2 major modifiable risk factors for chronic disease and premature death in the United States (1), yet most Americans are not meeting national guidelines in these important health areas (2,3). A growing body of evidence emphasizes the importance of the built environment in shaping physical activity and diet (4–9). Built environment features that have been associated with physical activity include proximity to parks, access to trails and recreational facilities, mixed land use, walkability, bikeability, accessible destinations, and opportunities for active transit (4,6–8,10,11). Studies examining the food environment have focused on availability of healthy foods in a community, most often measured by access to grocery stores or the density of fast-food restaurants as influences on dietary consumption (9).

Rural populations suffer a disproportionate burden of chronic disease (12).They also tend to have less healthy diets and are more sedentary than their urban counterparts (13,14). With fewer places within easy walking distance, rural residents are more dependent on cars (15) and walk less (16). Most research examining the relationship between built environment and health have focused on nonrural settings. In addition, there are few assessment tools designed for rural settings (6).

This article reports on the use of and results from an electronic tablet-based community assessment tool, the Stanford Healthy Neighborhood Discovery Tool (hereafter referred to as the Discovery Tool), to conduct built environment audits in rural settings. The primary objective of this qualitative study was to evaluate the usefulness of the tool in identifying barriers and facilitators to healthy eating and active living in a rural context. The second objective was to understand resident perspectives on community features and opportunities for improvement.

## Methods

**Figure1:**
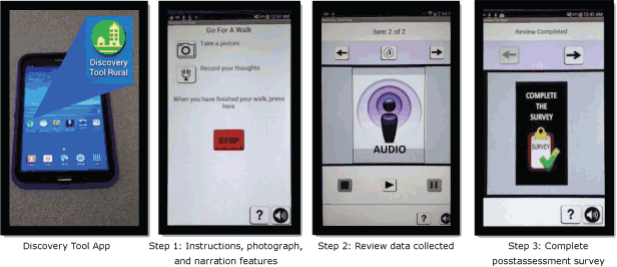
The Stanford Healthy Neighborhood Discovery Tool installed on a tablet and showing 3 steps for using the tool: 1) instructions for use and prompts for capturing photographs and narration, 2) review of the data collected, and 3) postassessment survey.

The Discovery Tool is a handheld electronic tablet-based assessment tool that records photographs, audio narratives, and walking routes (Figure 1) to help residents characterize built environment features in their communities (17). It was developed through a community-based participatory research approach (18) and extends the Photovoice methodology (19) by leveraging GPS technology to geocode data. It also includes a built-in postassessment survey to gather demographic characteristics of users and additional community-level information (Post-Assessment Survey, Appendix). 

We used the Discovery Tool (downloaded on Samsung 8 GB Galaxy Tab 3 Multi-Touch 7.0 tablets) in 4 rural communities in upstate New York from October 2013 through May 2014. For the purpose of our study, rural was defined as a town population with fewer than 10,000 people and a Rural Urban Commuting Area (RUCA, version 2.0) code of 4 or greater (http://depts.washington.edu/uwruca/ruca-codes.php). In each community, we partnered with a Cornell Cooperative Extension (CCE) educator who was trained to use the technology. Each CCE educator recruited participants from the general community through flyers, electronic lists, and direct contact. To ensure diverse representation of the sample, we stratified participants by age (40–64 y and ≥65 y) and sex and assigned each community 1 age–sex stratum. Adults who matched our community-specific age and sex stratification and resided in 1 of the 4 communities were eligible to take part in this study.

Participation in this qualitative study included 3 activities that each lasted approximately 60 to 75 minutes. The first 2 activities involved using the Discovery Tool to document resident-perceived barriers and facilitators to active living (Walk 1, October 2013) and healthy eating (Walk 2, April 2014) in the community. The Discovery Tool has been used to assess both the physical activity and food environments in urban settings previously (17,20). We determined on the basis of the prior audit work of 2 of the authors (R.A.S. and L.M.C.) and input from community partners that all walks would begin at a central location in each of the 4 communities. Participants were accompanied by their community’s CCE educator for both walks and could travel any route they chose. CCE educators silently followed each participant on individual walks to provide support in the event a technical question or problem arose. Participants were asked to capture aspects of their communities that made it easier or more difficult to be physically active (Walk 1) or eat healthfully (Walk 2), and we encouraged participants to pair photographs and audio narratives. At the end of the walks, each participant completed the postassessment survey on the tablet, and CCE educators completed a paper-based observation form. Observation forms were created for both walks as a way for the CCE educators to share key observations. Additional survey questions about the participants’ experiences were included as part of the observation form and administered by the CCE educator (Observation Form, Walk 1; Observation Form, Walk 2, Appendix). The third activity was a focus group in each community conducted in May 2014 by L.M.C. The topic guide for the focus group sessions was designed to gather feedback on use of the Discovery Tool in rural settings and to elicit discussion on experiences and opinions regarding active living and access to healthy foods in the community (Focus Group Guide, Appendix). Participants provided written informed consent and were compensated with a $75 gift card. The study protocol and all materials were reviewed and approved by the Cornell University Institutional Review Board.

Research assistants transcribed the audio narratives and recordings of the focus groups verbatim. Transcripts were coded by L.M.C. and J.A.G. in NVivo, version 10 (QSR International Pty Ltd), by using a framework based on the main research questions and emergent themes, with a subset of double coding and agreement checks by E.H.M. Survey data were analyzed by L.M.C. using SPSS version 21 (IBM Corp). Photos were used to contextualize audio narratives but were not independently coded.

## Results

### Participants

Twenty-four adults, 6 per community, participated in the study (Table 1). Mean age of the sample was 69.4 years (standard deviation [SD], 13.2 y). Population size of the 4 towns ranged from 2,206 to 6,625 residents. All participants self-identified as white and 1 identified as Hispanic/Latino, a racial/ethnic composition similar to that of the participating communities. Women made up 58.3% of the sample; 54.2% had a college degree, 43.5% had no previous experience with touchscreen technology, and 3 (12.5%) reported using an assistive walking device.

**Table 1:** Characteristics of Rural New York Communities and Participants, the Stanford Healthy Neighborhood Discovery Tool Project, New York State, October 2013–May 2014

Characteristic^a^	Community A (n = 6)	Community B (n = 6)	Community C (n = 6)	Community D (n = 6)	Total (n = 24)
**Participants**					
**Age, mean (SD)**	53.8 (8.2)	70.8 (7.7)	59.3 (14.7)	75.5 (9.8)	64.9 (13.2)
**Sex**					
Female	6	3	0	5	14
Male	0	3	6	1	10
**Race**					
White	6	6	6	6	24
**Ethnicity**					
Hispanic or Latino	0	0	0	1	1
**Highest level of education**					
Some high school	0	0	0	1	1
Completed high school	1	0	1	3	5
Some college	2	1	1	1	5
Completed college	3	5	4	1	13
**Uses assistive walking device**	0	0	1	2	3
**How far do you live from where you started the walk? **					
<1 mile	2	4	5	3	14
1−2 miles	0	2	1	1	4
>2 miles	4	0	0	2	6
**No previous experience with touch-screen technology**	2	2	1	5 **b**	10
**Community**					
Town population	5,576	2,206	6,625	4,048	—
County population density, people per square mile	31	45	52	56	—
Median annual household income, $	37,063	40,938	46,889	43,115	—
Race, % white	97.0	96.9	92.3	99.1	—

Abbreviation: SD, standard deviation; —, not applicable.

a Values are n unless otherwise indicated.

b Data for this category were missing from 1 participant in Community D (n = 5).

### Built environment features

The Discovery Tool captured 462 photographs and 454 audio narratives. On each outing, participants walked an average of 50.1 minutes (SD, 15.9) and an average of 0.99 miles (SD, 0.52); they recorded an average of 9.8 photographs (SD, 5.4) and 9.7 (SD, 5.0) audio narratives. Participants often captured both positive and negative features of the built environment in a single paired photograph and audio recording.

**Figure 2:**
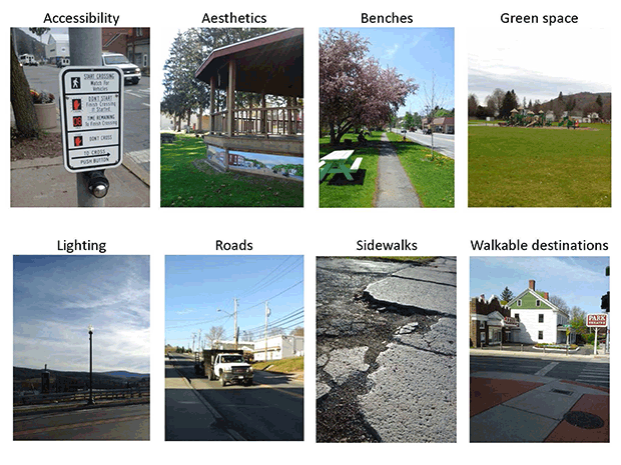
Eight common features related to active living identified and photographed by participants. Photos were used to contextualize audio narratives but were not independently coded.

Eight common features related to active living emerged from the data (Table 2) (Figure 2). The most frequently captured features were characteristics of roads, sidewalks, and walkable destinations. Many participants (80.8%) felt local roads were unsafe or lacked well-labeled or enforced crosswalks that would facilitate walking. Both positive and negative aspects of sidewalks were recorded. Well-maintained sidewalks connecting residential areas with schools and the town center were considered a facilitator to active living by 57.7% of participants; absent, cracked, or uneven sidewalks were cited as barriers to community walkability by 73.1% of participants. Shops, entertainment venues, and community services (eg, post office, churches) were recorded as walkable destinations. However, several participants (42.3%) photographed vacant buildings and lots and described their communities as in decline and trending toward fewer local destinations and a less aesthetically pleasing atmosphere.

**Table 2:** Description of Environmental Features Related to Physical Activity Identified Through Community Assessments Conducted in 4 Rural Communities by Using the Stanford Healthy Neighborhood Discovery Tool and Follow-Up Focus Groups, New York State, October 2013–May 2014

Coded Feature and Description	Quotations (+ or −)
**Accessibility:** Features that impact accessibility and safety for people with impaired mobility	“[This is] an intersection that’s handicap accessible . . . the curbs are cut down so that you can roll across with a [manual] wheel chair or motorized wheel chair. This makes it very easy for people to access our main street [on] both sides.” (+)
**Aesthetics:** Features that improve or worsen the atmosphere, such as landscaping	“We thank God for our lovely volunteers that volunteer their time to plant these flowers and stuff so that when we are walking around the park, we have nice things to look at.” (+)
**Benches:** Benches, picnic tables, or other seating positioned around town	“Okay here’s a couple more benches that we found in this little park here. [It’s] another place to have a nice rest, [if] you’re doing some walking in the village and you want to take a little break.” (+)
**Green spaces:** Outdoor spaces suitable for walking, playing, or exercising, including trails, parks, forests, sports fields, and outdoor pools	“This is a picture of a nice walking path from [this town] to the backside of [the] elementary school to the playground. Easy access and scenic.” (+) “[This] fitness trail would not be extremely safe for [the] elderly. The conditions of the trail are actually more of a hike with uneven ground.” (−)
**Lighting:** Outdoor lighting	“I really like these lamp posts. They really enhance being able to walk with your family members in the evening. You can see, it’s well lit, and they’re pretty.” (+) “What I observe when I go through town is not only in the daytime but at nighttime is that we need more lighting, more street lights, to see the sidewalks better.” (−)
**Roads:** Roads and crosswalks; traffic safety concerns including speed of traffic, dust, and noise pollution	“When you’re walking – especially on Main Street and east and west of Main Street – there’s lots of traffic, lots of noise, [and] lots of dust in places where the cars go faster and it makes it unpleasant to walk. You can’t talk if you’re with somebody . . . ” (−) “Very few crosswalks, even the ones that are labeled, don’t really have any other visual signs that say stop for pedestrians in this walkway. So, pretty dangerous actually.” (−)
**Sidewalks:** Presence, absence, and condition of sidewalks	“[The sidewalks are a] bad situation. An older person could fall over that and really get hurt. Or anybody of any age could get hurt on that because it’s a pretty good size height there on that sidewalk that has risen up so I think it could be fixed.” (−) “On this street, the sidewalk just ends so you have to cross the street to get to a sidewalk.” (−) “As you can see, everything is very level, very straight. The sidewalks are all new.” (+)
**Walkable destinations:** Local destinations for shopping, entertainment, or services	“This is a picture of a classic movie theater, very old fashioned in our community. Lots of people go here and walk here from around the village.” (+) “Empty storefronts are somewhat of a blight on a small town like this because there just aren’t enough outlets for people in town because of the larger supermarkets and things like that beyond town. So in town, buildings remain empty.” (−)

Abbreviations: +, active living facilitators; − barriers to active living.

**Figure 3:**
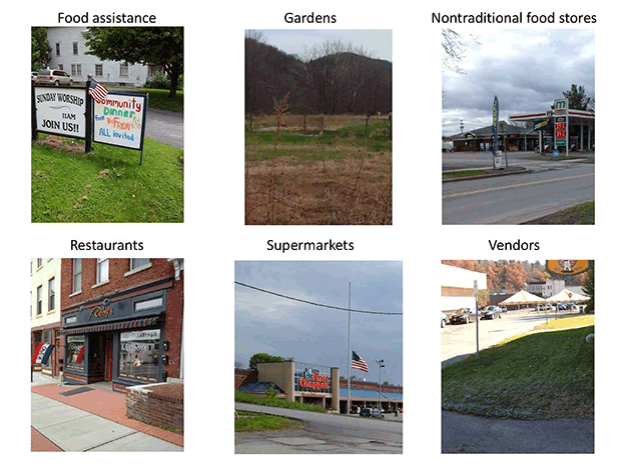
Six common features of the food environment identified and photographed by participants. Photos were used to contextualize audio narratives but were not independently coded.

Participants captured 6 common features of the food environment (Table 3) (Figure 3). The most frequently documented features were restaurants, nontraditional food stores (eg, convenience stores, pharmacies), and supermarkets, usually with reference to the cost, quality, and selection of healthy foods available. Large food purchases were sometimes made at supermarkets outside of town to access a wider range of foods at potentially lower prices, with nontraditional food stores in town used for staples such as milk, eggs, and bread and “quick, convenient snacky type things.” However, some participants felt that food shopping outside of town was unnecessary or uneconomical when the cost of gas was considered and noted that smaller nontraditional food stores in town often priced their staple items competitively and offered regular specials. A subset of participants (34.6%) discussed farmers markets, farm stands, and gardens as sources of fresh produce in season. Thus, a combination of nontraditional food stores and locally grown foods were perceived to be important features, both positive and negative, of the food environment in these rural towns.

**Table 3:** Description of Environmental Features Related to Healthy Eating Identified Through Community Assessments Conducted in 4 Rural Communities in New York by Using the Stanford Healthy Neighborhood Discovery Tool and Follow-up Focus Groups, October 2013 – May 2014

Coded Feature and Description	Quotations (+ or −)
**Food assistance:** Programs that provide free or subsidized food, such as community food pantries, Meals on Wheels, and federal nutrition assistance programs	“This is the Methodist church. We took a picture of this because this is where the food pantry is located and it’s also a site for WIC [the Special Supplemental Nutrition Program for Women, Infants, and Children]. I think they come maybe once a month.” (+)
**Gardens:** Home and community gardens	“This is a picture of [town’s] community garden where many of the friends and neighbors participate and plant different vegetables that they swap and share with each other and they also donate to community food pantry.” (+)
**Nontraditional food stores:** Pharmacies, convenience stores, gas station food marts, dollar stores, and other nontraditional venues for food sales	“It’s the only pharmacy they have here. I frequent this quite often. I get staples like milk, eggs, and, you know, greeting cards. That sort of thing. It’s a very nice place. The people are very friendly, very neat. It does good business and I’m glad to have it here.” (+) “[I] have never seen fresh produce of any kind in here. You know just very quick, convenient snacky type things, a lot of candy and high sugar beverages. Not a place where I would do a large amount of shopping.” (−)
**Restaurants:** Full-service and fast-food restaurants, cafes, and coffee shops	“And it’s a pretty good restaurant where they do have a variety of different menu options including a nice salad bar.” (+) “This is family restaurant that you can get healthy food at. I go here once a week. One of my friends that I dine with is gluten free so she has to be very careful what she eats. And they do have very healthy choices on the menu. But not only gluten free, regular vegetarian, Weight Watchers.” (+) “And down [street] — which is right of Main Street — is what I just took a picture of: sort of a bar and a sports bar. It has some food. Most of it’s unhealthy and, of course, [it serves] lots of alcohol.” (−)
**Supermarkets:** Grocery stores and supermarkets	“This is the [supermarket]. Really the only supermarket in the entire town but pretty much anything that one needs can be purchased there.” (+) “Only one grocery store so we’re limited to what they have. The prices are actually comparable for fresh fruits and vegetables to [other larger towns], as far as fresh produce.” (+/−) “I would like to see a new grocery store come in for some more competition, maybe lower prices.” (−)
**Vendors:** Farmers markets and seasonal food stands	“This is a picture of the lawn in front of [hospital] where they will be having a farmers market in the summer starting in July through September — first and third of the month. All produce is from local farmers.” (+) “This is a photo of the area where the farmers market is in the summer. A big tent is put up and speed bumps are put in the parking lot with directional signs. The biggest challenge is getting farmers.” (+/−)

Abbreviations: +, healthy eating facilitators; − barriers to healthy eating.

### Use of the Discovery Tool in a rural setting

Overall, participants described the Discovery Tool as easy, interesting, and fun to use. In particular, participants enjoyed the ability to capture their thoughts and observations in real time and link photos with audio narratives: “It was pretty self-explanatory for someone who wasn’t familiar with a tablet.”

Starting from a central location was considered expedient in a rural context for making assessments. Participants frequently described driving to local amenities and destinations. Participants who did not live near town felt it would have been different and difficult to complete their walks if they had begun at home. Some participants also expressed that capturing the experiences of community members who live within and outside of town increased the representativeness of data collected: “It would have been a long and difficult walk on a busy county highway [if I had started at home]”; “I think for those of us that live in that 5-minute area at the center of town, we have a different perception of what’s going on in the village than maybe people on the fringes.”

### Awareness and opportunities for change

Participants described how the Discovery Tool helped them to become more aware of environmental features in their communities. The experience of purposefully walking through the community and documenting the built environment with photos and audio narratives often gave participants a new perspective on barriers and facilitators to active living and healthy eating: “I appreciated the chance to walk around. Some of those restaurants I had never been [to] before. They have a new bakery. They’ve got a nice breakfast and lunch menu. I hadn’t been in there, so actually I went there last week because of the walk”; “[The Discovery Tool] really helped me to understand the community a lot better and open my eyes to a lot of the things that are going [on] around here.”

Overall, participants felt that the Discovery Tool was helpful for identifying and prioritizing opportunities for community improvement projects. Multiple suggestions were provided for situations in which the tool might be useful, including familiarizing residents with available resources and bringing concerns to local leadership: “I think it might be helpful if you were going to a village board meeting . . . or go to planning boards or go to individuals that might be making decisions about fixing the sidewalks or putting in a trail”; “It’s a tool that, used appropriately, could really help us to magnify something in this village that we might want to focus on.”

## Discussion

This study identified many of the themes that have been identified through other environmental assessments in rural settings (6,21) but with a resident-based perspective on what such environmental elements mean in residents’ daily lives. Not surprisingly, the food environment was characterized according to the selection, prices, and quality of foods offered at local supermarkets, nontraditional food stores, restaurants, farmers markets, gardens, food banks, and soup kitchens. Participants indicated that the following factors are likely to affect physical activity: condition of roads and sidewalks; the existence of walkable destinations and greenspaces; and the presence of good lighting, benches, aesthetic features, and handicapped-accessible pathways. Preliminary findings from other studies that have used the Discovery Tool in urban settings in California, Mexico, and Israel, indicate both differences and similarities in the barriers and facilitators to healthy active living noted by urban participants compared with rural participants (22–24). As did participants in this study, participants in urban settings noted the quality of roads and sidewalks, destinations to visit, parks, aesthetics, and mobility and access issues as important contributors to healthy active living. Differences included observations about trash, personal safety, and street features noted by urban participants but not by rural participants and the presence of benches and places to rest while walking noted by rural participants and not by urban participants. This is the first study that we are aware of that has used this type of electronic, science-oriented crowdsourcing tool for assessing built environments in a rural setting.

Our study demonstrated that the Discovery Tool may be well suited to capture residents’ perspectives on environmental features that affect active living and healthy eating in rural contexts. Despite a lack of prior experience, participants felt that the electronic tablet-based tool was easy to use, engaging, and helpful in characterizing the built environment. We found that the tool could be used by rural residents with minimal instruction, regardless of age or touch-screen literacy. Responding to feedback following Walk 1, we made minor modifications to improve usability, including adjusting the brightness and sleep mode settings, adding antiglare screen protectors, and adding protective cases. These types of modifications may have been particularly helpful for rural seniors, who often experience considerable barriers to computer use (25).

The interactive nature of this electronic tablet–based technology is an asset for community-based participatory research because it involves residents in the assessment process and allows them to capture their environment in their own images and words. The Discovery Tool, or similar tools, may complement existing paper-based audit tools by helping identify environmental features that have been overlooked in other assessments. Data gathered could be harnessed to help build local consensus and catalyze community change. Community-specific summary reports were generated from the assessment data collected in this study, and 1 participating community is actively leveraging insight gained through the assessment to conduct a resident-led project to change the built environment by increasing physical activity options for older adults.

Limitations include the small sample size in this first-generation study, and its focus on upstate New York communities. However, the diversity of the sample of older adults in terms of age, sex, mobility, and familiarity with touch-screen technology is a study strength and suggests that this type of technology and approach may be suitable for use by rural residents. Further testing and research using a larger and racially diverse sample and geographically diverse locations is warranted. Furthermore, feedback from the participants may not reflect the everyday experiences and perceptions of a community because the use of this innovative tool may prompt identification of barriers and facilitators in the built environment that would not normally be identified by the lay community member. Strengths of this study are that it is one of the first built environment assessments to include rural residents 40 years of age or older (6), to include multiple walks to capture in detail both active living and healthy eating environmental elements, and to provide a rich data set through a combination of photos, audio recordings, maps, survey data, and follow-up focus groups.

Participatory, electronic tablet–based (or other electronic) tools may be particularly suited for research applications in identifying and catalyzing resident-led change in rural food and physical activity environments.

## Appendix Postassessment Survey, Observational Forms, and Focus Group Guide.

This file is available for download as an Adobe Acrobat Reader document.
