# Supplementary material for: Rural Food and Physical Activity Assessment Using an Electronic Tablet-Based Application, New York, 2013–2014
Source: Prev Chronic Dis. 2015 Jul 2;12:E102. doi: 10.5888/pcd12.150147 (PMC4492214; doi:10.5888/pcd12.150147)
Supplement: Supplementary file 1 [file 15_0147_Appendix.pdf]

Post-Assessment Survey **Appendix**

Post-Assessment Survey questions completed on tablet

1) What is your age? (Pick only one)
   ☐ 17 years or younger
   ☐ 18 to 35 years
   ☐ 36 to 49 years
   ☐ 50 to 64 years
   ☐ 65 to 89 years
   ☐ 90+ years

2) What is your gender? (Pick only one)
   ☐ Female
   ☐ Male

3) What is your ethnicity? (Pick only one)
   ☐ Hispanic or Latino
   ☐ Not Hispanic or Latino
   ☐ Prefer not to answer

4) What is your race? (Pick only one)
   ☐ American Indian or Alaskan Native
   ☐ Asian
   ☐ Black or African American
   ☐ Native Hawaiian or Other Pacific Islander
   ☐ White
   ☐ Other

5) What is your highest education level completed? (Pick only one)
   ☐ Less than 7th grade
   ☐ Junior high/middle school
   ☐ Some high school
   ☐ Completed high school
   ☐ Some college or vocational training
   ☐ Completed college or university

6) What statement best describes where you live?
   ☐ Urban
   ☐ Suburban
   ☐ Rural (I live in a rural town/village)
   ☐ Rural (I live outside of a rural town/village)

7) How far is the town center from where you live?
   ☐ Less than a mile
   ☐ 1-5 miles
   ☐ More than 5 miles

8) Do you regularly use a cane, walker, scooter, or another assistive device when you go outside of your home?
   ☐ Yes
   ☐ No

Post-Assessment Survey questions completed on tablet

9) What types of transportation do you normally use? (Check all that apply)

☐ Bus
☐ Train or subway
☐ Shuttle van
☐ I drive myself
☐ Someone else drives me
☐ Other
☐ None of these

10) From where you live, how difficult/easy is it for you to **walk or bike** to places you want to go (e.g. store, church, library, bank)?

☐ Very difficult
☐ Difficult
☐ Not that difficult
☐ Easy
☐ Very easy

11) What makes it difficult to walk or bike to businesses and other destinations you visit? (check one or more)

☐ I live far away from these services and amenities
☐ There is a lack of sidewalks and/or bicycle paths
☐ The condition of the sidewalks and/or bicycle paths are poor
☐ I fear that people will think I walk/cycle because I don't have a car or lost my license
☐ I have difficulty walking or biking due to personal health conditions
☐ Other
☐ None

12) What types of places do you walk to? (Check all that apply)

☐ Food stores
☐ Public services
☐ Commercial services or stores
☐ Recreational facilities
☐ Other
☐ None of the above

13) If you checked "**Food Store**," what type of food store do you walk to? (Check all that apply)

☐ Bakery
☐ Convenience store
☐ Supermarket
☐ Small fruits and vegetable store
☐ Fast food restaurant
☐ Non fast food restaurant
☐ Coffee shop
☐ Other
☐ None of the above

Post-Assessment Survey questions completed on tablet

14) If you checked "**Public Services**," what type of public services do you walk to? (Check all that apply)
☐ Post office
☐ Library
☐ Schools
☐ Senior center
☐ Health care clinic
☐ Other
☐ None of the above

15) If you checked "**Commercial services or stores**," what type of commercial services or stores do you walk to? (Check all that apply)
☐ Pharmacy/drug store
☐ Hardware store
☐ Laundry/dry cleaner
☐ Clothing store
☐ Book store
☐ Bank/Credit union
☐ Video store
☐ Salon/barber
☐ Paid/volunteer work
☐ Other
☐ None of the above

16) If you checked **"Recreational facilities**," what type of recreational facilities do you walk to? (Check all that apply)
☐ Park
☐ Recreation Center
☐ Gym/Fitness facility
☐ Other
☐ None of the above

17) Is availability a barrier to purchasing healthy foods such as fresh fruits and vegetables in your community? (check one or more)
☐ Yes
☐ No
☐ I would have to drive 10+ miles to reach them

18) Is cost a barrier to purchasing healthy foods such as fresh fruits and vegetables in your community? (check one or more)
☐ Yes
☐ No
☐ I would have to drive 10+ miles to reach the more affordable options

19) What type of residence do you live in? (Pick only one)
☐ Single family house
☐ Multi-family house
☐ Apartment/Condominium
☐ Other

Post-Assessment Survey **Appendix**

Post-Assessment Survey questions completed on tablet

20) Is your residence for seniors only?
    ☐ Yes
    ☐ No
21) What was the purpose of your walk today? (Pick only one)
    ☐ Get to public transportation
    ☐ Get to a particular destination or run an errand
    ☐ Pleasure or leisure (no particular destination)
    ☐ Exercise
    ☐ Other
    ☐ None of the above

22) Were there sidewalks on your walk today?
    ☐ Yes
    ☐ No

23) Were there cracks, un-evenness, or holes in the sidewalks on your walk today?
    ☐ Yes
    ☐ No

24) Were there ramps in the sidewalks at street corners as pictured below on your walk today?
    ☐ Yes
    ☐ No

25) Was there space between the sidewalk and the street as pictured below on your walk today?
    ☐ Yes
    ☐ No

26) Were there stray animals or unleashed dogs on your walk today?
    ☐ Yes
    ☐ No

27) Did you see or smell air pollution on your walk today?
    ☐ Yes
    ☐ No

28) Did you hear excessive noise like trains, construction, or loud factories on your walk today?
    ☐ Yes
    ☐ No

29) Were there people on your walk today that made you feel unsafe? (Check all that apply)
    ☐ Gangs
    ☐ Youth
    ☐ Unemployed
    ☐ Homeless
    ☐ People under the influence of drugs or alcohol
    ☐ Other
    ☐ None of the above

Post-Assessment Survey questions completed on tablet

30) Did you see any of these things on your walk today? (Check all that apply)
    - ☐ Bottles or broken glass
    - ☐ Cigarette butts
    - ☐ Condoms
    - ☐ Needles or syringes
    - ☐ Garbage or litter
    - ☐ Graffiti
    - ☐ Broken windows
    - ☐ Abandoned buildings
    - ☐ None of the above

31) Did you see any of these street features on your walk today? (Check all that apply)
    - ☐ Speed bumps
    - ☐ Stop signs
    - ☐ Traffic lights
    - ☐ Median island
    - ☐ Traffic circle or roundabout
    - ☐ Electronic signs that display traffic speeds
    - ☐ None of these

Observation Form, Walk 1 **Appendix**

Date of walk:__ __ / __ __ / __ __ Observer: _______________

Tablet Number: _________________

Start time: _______________________ End Time: ________________________

Name of Participant: ____

Address: ____

Contact telephone numbers: ____

Gender ☐ Male ☐ Female Date of Birth ____________________________________

Town: ______________________________ County: ____

Other people present (e.g. spouse, friend, etc.):_______________________ _______________________

Was the walk interrupted by a phone call, medical concern, or some other competing issue?☐ Yes ☐ No

Did the participant seem rushed on the walk? ☐ Yes ☐ No

How engaged was the participant in this task?

Not at all engaged		Somewhat engaged		Very engaged
1	2	3	4	5

Observations: ____

____

____

____

____

Questions to ask the participant

After your experience today, what do you think are the main issues that affect the opportunities people living in your community have to lead active healthy lives?

____

____

How far do you live from where the walk started today? ______________________________________

What are the reasons why you come to this town/location, and how often do you do so?

____

____

Do you walk or drive to come to this location? ____

Would using the Discovery Tool be different if you had used it directly from your home? How?

____

____

Observation Form, Walk 1 **Appendix**

Discovery Tool					
**1. How interesting (cool/fun) was it to wear or carry the device?**					
Not at all<br>1	Not very<br>2	A little<br>3	More or less<br>4	Somewhat<br>5	Very<br>6
**2. How comfortable was the device to wear on your body or carry?**					
Not at all<br>1	Not very<br>2	A little<br>3	More or less<br>4	Somewhat<br>5	Very<br>6
**3. How nervous did you feel wearing or carrying the device?**					
Not at all<br>1	Not very<br>2	A little<br>3	More or less<br>4	Somewhat<br>5	Very<br>6
**4. How self-conscious did you feel wearing or carrying the device?**					
Not at all<br>1	Not very<br>2	A little<br>3	More or less<br>4	Somewhat<br>5	Very<br>6
**5. How dangerous did it feel to wear or carry the device?**					
Not at all<br>1	Not very<br>2	A little<br>3	More or less<br>4	Somewhat<br>5	Very<br>6
**6. How much did you enjoy using the device to record information about your neighborhood environment?**					
Not at all<br>1	Not very<br>2	A little<br>3	More or less<br>4	Somewhat<br>5	Very<br>6
**7. How likely would you feel recommending to your friends that they use the device?**					
Not at all<br>1	Not very<br>2	A little<br>3	More or less<br>4	Somewhat<br>5	Very<br>6
**8. If asked to do so, how likely would you be to use this device again?**					
Not at all<br>1	Not very<br>2	A little<br>3	More or less<br>4	Somewhat<br>5	Very<br>6
**9. If asked to do so, would you be willing to use this device for a longer period of time?**					
Not at all<br>1	Not very<br>2	A little<br>3	More or less<br>4	Somewhat<br>5	Very<br>6

Discovery Tool
10. What did you like about using the device?
11. What did you NOT like about using the device?
12. If there was one thing you would change about using the device, what would that be?
13. How do you think the images from the device might be useful to you?
14. What did using this device help you to notice about your neighborhood that you might not have noticed before?
15. The Discovery Tool was intended to use in urban communities. When using the tool, was there anything you felt the tool was unable to capture?
16. Anything else you would like to tell us about the device?

Observation Form, Walk 2 **Appendix**

Date of walk: __ __ / __ __ / __ __ Observer: _______________

Tablet Number: _________________

Start time: ________________________ End Time: _________________________

Name of Participant: ____

County: ____

Observations: ____

____

____

____

____

____

Questions to ask the participant

In the Fall, you completed your first walk using the Discovery Tool. Prior to engaging in this walk, did you have any experience using an electronic touch-screen device similar to this tablet (this may include a smart phone like an IPhone or a tablet like an IPad)?

____Yes

____No

Are you *currently* involved in any type of civic engagement activities in your town/village? This could include: member of the town board, community volunteer, or any similar community building roles?

____Yes (please explain below)

____No

____

____

Have you *previously* been involved in any type of civic engagement activities in your town/village? This could include: member of the town board, community volunteer, or any similar community building roles?

____Yes (please explain below)

____No

____

____

(A) INTRODUCTION

First, I would like to thank everyone for taking part in the community assessment. We are very excited to have had the opportunity to use the Discovery Tool in your community. Today, we are going to discuss your experiences and findings from your community assessment.

In the Fall, you were asked to use the Discovery Tool to capture aspects of your community that make it easier or more difficult to be physically active. A few weeks ago, you were given a second opportunity to capture these items as well as features of the food environment in your community.

(B) STRENGTHS TO ACTIVE LIVING IN YOUR COMMUNITY

We would like to begin by discussing some of the strengths or assets to active living within your community.

1.) Does anyone want to volunteer any strengths you identified in your community that promote physical activity?

[Probes]
- What did you capture using the Discovery Tool?
- Were these new findings or were you already aware of these discoveries?
- What services/features does your community offer to promote physical activity?

(C) BARRIERS TO ACTIVE LIVING IN YOUR COMMUNITY

Now we would like to focus on some of the barriers that you identified that discourage physical activity within your community.

2.) Does anyone want to volunteer any barriers you identified in your community that prevents or discourages physical activity?

[Probes]
- What did you capture using the Discovery Tool?
- Were these new findings or were you already aware of these discoveries?
- What services/features does your community lack? What services/features would you like to have in your community to promote/enhance physical activity?

We are now going to transition into talking about some strengths/assets that your community possesses related to healthy eating. On your second walk, you were asked to capture strengths and weaknesses to eating healthy in your community by documenting the food outlets in your town/village.

(D) STRENGTHS TO HEALTHY EATING IN YOUR COMMUNITY

3.) Does anyone want to volunteer any strengths that you identified in your community for healthy eating?

[Probes]

- What did you capture using the Discovery Tool?
- Were these new findings or were you already aware of these discoveries?
- What services/features does your community offer to promote healthy eating (e.g. farmer's markets, community gardens, etc.)?

(E) BARRIERS TO HEALTHY EATING IN YOUR COMMUNITY

Now we would like to focus on some of the barriers that you identified that discourage healthy eating within your community.

4.) Does anyone want to volunteer any barriers you identified in your community for healthy eating?

[Probes]

- What did you capture using the Discovery Tool?
- Were these new findings or were you already aware of these discoveries?
- What services/features does your community lack? What services/features would you like to have in your community to promote healthy eating (e.g. farmer's markets, community gardens, etc.)?

5.) We asked participants, "Is availability a barrier to purchasing healthy foods such as fresh fruits and vegetables in your community?" Please tell me more about this.

6.) We asked participants, "Is cost a barrier to purchasing healthy foods such as fresh fruits and vegetables in your community?" Please tell me more about this.

(F) DISCOVERY TOOL USE - GENERAL

Now I would like to ask you about the pros/cons to using the Discovery Tool to capture the strengths and weaknesses of your community related to healthy eating and active living.

7.) What did you like about using the Discovery Tool?

8.) What didn't you like about using the Discovery Tool?

9.) If an outsider looked at your pictures and audio recordings do you feel that he/she would be able to accurately assess the opportunities and barriers that your community has to be **physically active**?

[Probes]

- What were you not able to capture using the Discovery Tool?

10.) If an outsider looked at your pictures and audio recordings do you feel that he/she would be able to accurately assess the opportunities and barriers that your community has to access **healthy food**?

[Probes]

- What were you not able to capture using the Discovery Tool?

(F) DISCOVERY TOOL USE – RURAL STARTING POINT

The Discovery Tool was designed for use in urban neighborhoods. We adapted the instructions in the Discovery Tool to encourage participants in rural communities to start their walk in the town center. Instructions now state "If you reside in a rural location, we would like you to begin your walk wherever you consider the "center of town." This could include the church, library, town hall, post office, hardware/general store, coffee shop, or any other location that you and others in your community may likely identify as the central location or area."

11.) Are these instructions clear?

12.) Approximately 40% of participants reported that using the Discovery Tool would be different if used from their home? What differences would your observe or capture?

13.) Would it be possible to use the Discovery Tool from your home? Why or why not?

14.) Do you agree with our decision to have participants start their walk from the town center? Why or why not?

(G) CONCLUSION

Are there any other comments that you would like to share related to healthy eating and active living in your community environment?

Thank you for participating in the community audit and focus group.
